# Antitumor Effects of Laminaria Extract Fucoxanthin on Lung Cancer

**DOI:** 10.3390/md15020039

**Published:** 2017-02-15

**Authors:** ChengHan Mei, ShunChang Zhou, Lin Zhu, JiaXiong Ming, FanDian Zeng, Rong Xu

**Affiliations:** 1 Department of Pharmacology, School of Basic Medicine, Tongji Medical College, Huazhong University of Science and Technology, Wuhan 430030, China; ckklmch@sina.cn (C.M.); zhul_1024@sina.cn (L.Z.); jxminghust@163.com (J.M.); fdzeng@163.com (F.Z.); 2 Department of Laboratory Animal, Tongji Medical College, Huazhong University of Science and Technology, Wuhan 430030, China; schzhou@sohu.com

**Keywords:** fucoxanthin, non-small-cell lung cancer, cell cycle arrest, apoptosis

## Abstract

Lung cancer is the leading cause of cancer mortality worldwide and non-small-cell lung cancer (NSCLC) is the most common type. Marine plants provide rich resources for anticancer drug discovery. Fucoxanthin (FX), a *Laminaria japonica* extract, has attracted great research interest for its antitumor activities. Accumulating evidence suggests anti-proliferative effects of FX on many cancer cell lines including NSCLCs, but the detailed mechanisms remain unclear. In the present investigation, we confirmed molecular mechanisms and in vivo anti-lung cancer effect of FX at the first time. Flow cytometry, real-time PCR, western blotting and immunohistochemistry revealed that FX arrested cell cycle and induced apoptosis by modulating expression of p53, p21, Fas, PUMA, Bcl-2 and caspase-3/8. These results show that FX is a potent marine drug for human non-small-cell lung cancer treatment.

## 1. Introduction

Lung cancer is currently the most prevalent malignant tumor worldwide and has the fastest rising incidence and mortality rate of all cancers [1]. Lung cancers are classified into small cell lung carcinoma (SCLC) and non-small cell lung carcinoma (NSCLC). The latter accounts 85% of all cases. Most of NSCLC cases have reached locally advanced or metastatic stage at the time of diagnosis. Unlike SCLC, average five-year survival rate of NSCLC is only 10%–15% [2]. The well-established platinum-based regimen can only bring a five-year absolute benefit of 5.4% [3]. Over the past 30 years, many potential therapeutic targets of lung cancer have been identified. Targeted therapy with epidermal growth factor receptor-tyrosine kinase inhibitors (EGFR-TKIs) have benefited many lung cancer patients, but they still suffer from drug resistance and compatibility [4,5]. To improve patients’ survival and the quality of life, novel approaches to NSCLC are of special interest.

Numerous effective anticancer drugs have been discovered from natural sources, but an important untapped resource in edible seaweeds remains to be exploited. Among them, *Laminaria japonica*, a brown alga, has a long history of use in the diets of Asian cultures. It has also been used as anti-thyroid tumor, edema, tuberculosis and beriberi drugs in traditional Chinese medicine [6]. FX is an oxygenated carotenoid available from multiple species of edible seaweeds, e.g., *Laminaria japonica*, *Undaria pinnatifida* and *Hijikia jusiformis* [7]. It has shown to possess many biological activities such as suppression of pre-adipocyte differentiation [8], anti-mutagenicity [9], anti-ocular inflammation and anti-oxidation [10]. Moreover, accumulating evidence has shown that FX has anti-cancer cancer cell effects on several cancer cell lines, including lung, leukemia, cutaneous, colon, liver, prostate and breast cancer [11,12,13,14,15,16,17]. While the in vivo anti-tumor effects and underlying molecular mechanisms for its anticancer activity are still unclear, we isolated FX from *Laminaria japonica* and investigated its anti-cancer effects in NSCLC cells and in tumor-bearing nude mice.

## 2. Results

### 2.1. FX Inhibits Proliferation in NSCLC Cells

**Figure 1:**
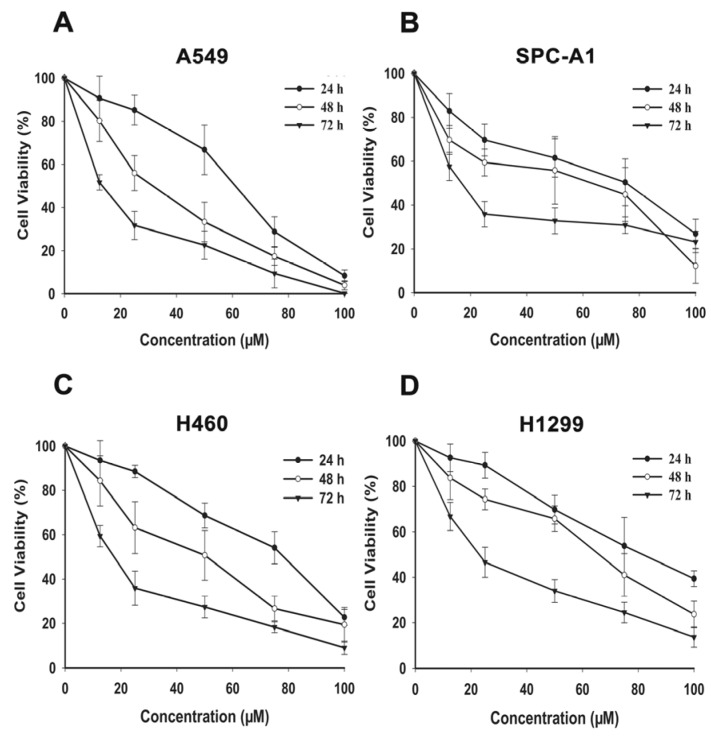
Effects of FX on proliferation of human NSCLC cells. Cytotoxicity of FX was assayed by CCK-8 method. A549 (**A**), SPC-A1 (**B**), H460 (**C**) and H1299 (**D**) cells were grown in a 96-well plate and exposed to various concentrations of FX (0, 12.5, 25, 50, 75 and 100 μM) for 24, 48 and 72 h. Data are presented as means ± SD (*n* = 3).

Human non-small cell lung cancer cells were treated with FX (12.5–100 μM) for 24, 48, or 72 h. Cell proliferations were significantly inhibited by FX (Figure 1). IC_50_ of NSCLC cells were presented in Table 1. The results demonstrated that FX exhibited a dose and time-dependent cytotoxicity effect on NSCLC cells.

### 2.2. FX Induces Cell Cycle Arrest in NSCLC Cells

**Figure 2:**
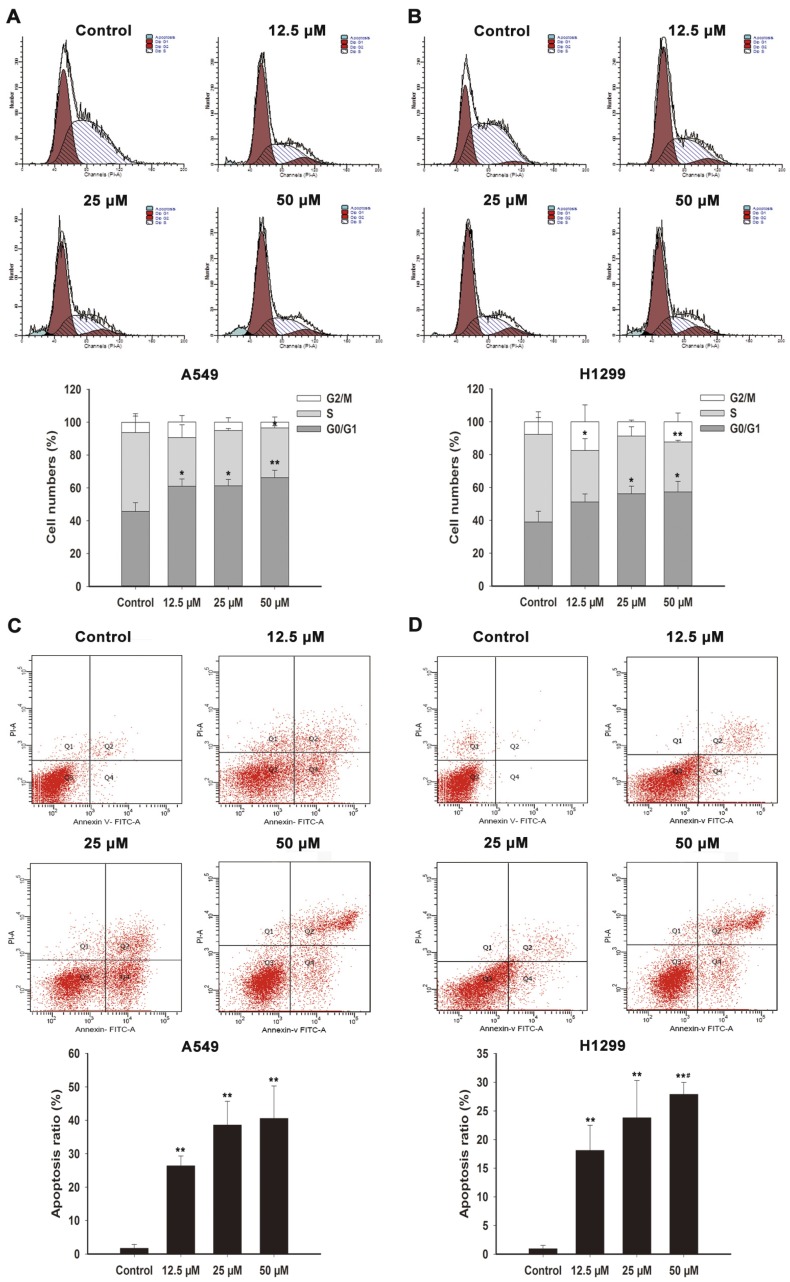
Induction of G_0_/G_1_ arrest and apoptosis by FX in A549 and H1299 cells. A549 (**A**) and H1299 (**B**) cells after the 48-h treatment of FX were stained with PI, then DNA content was analyzed by flow cytometry. G_0_/G_1_, S and G_2_/M indicate different cell cycle phases. Annexin V-FITC/PI staining assay of A549 (**C**) and H1299 (**D**) cells was analyzed by flow cytometry to detect apoptosis after FX treatment for 48 h and Q4 represent cells in apoptotic stage. Data are presented as means ± SD (*n* = 3). * *p* < 0.05, ** *p* < 0.01 compared to the control group, ^#^ *p* < 0.05, compared to the FX 12.5 μM treatment group.

To determine the mechanisms by which FX inhibited NSCLC cells proliferation, we first investigated the effects of FX on cell cycle progression. A549 and H1299 cells were treated with FX (12.5, 25 and 50 μM). After 48 h, the cells were analyzed by flow cytometry. As shown in Figure 2A,B and Table 2, treatment with FX increased the percentage of cells in the G_0_/G_1_ phase of the cell cycle and reduced the percentage of cells in the S phase. The G_0_/G_1_ arrest effect was concentration-dependent. 

### 2.3. Effects of FX on the Expression of Cell Cycle Regulatory Proteins in NSCLC Cells

**Figure 3:**
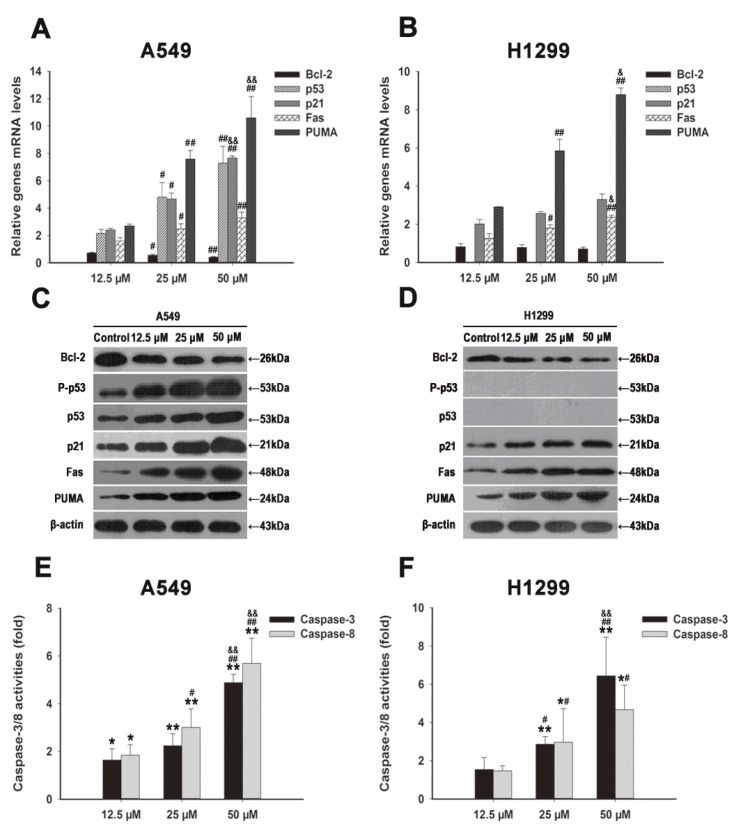
FX induced G_0_/G1 arrest and apoptosis through regulating p21^waf1/cip1^, p53, Bcl-2, PUMA and Fas. Total cell RNA was extracted from A549 (**A**) and H1299 (**B**) cells after treating with FX (0, 12.5, 25, 50, 75 and 100 μM) for 48 h. Corresponding mRNA levels of p53, p21^waf1/cip1^, Bcl-2, PUMA and Fas genes were determined using qRT-PCR. Results were shown as the relative expression ratio of genes in A549 and H1299 cells, respectively. Western blot analysis of cell cycle arrest and apoptosis related proteins: p53, P-p53, PUMA, p21^waf1/cip1^, Fas and Bcl-2, were performed after A549 (**C**) and H1299 (**D**) cells harvested after exposing to various concentrations of FX (0, 12.5, 25, 50 μM). Effects of FX on caspase-3/8 activities in A549 (**E**) and H1299 (**F**) cells were measured after 48 h of exposure similar to panel C/D by colorimetric method. When presented, means and standard deviations were obtained from three independent experiments. * *p* < 0.05, ** *p* < 0.01 compared to the control group; ^#^ *p* < 0.05, ^##^ *p* < 0.01 compared to the FX 12.5 μM group; ^&^ *p* < 0.05, ^&&^ *p* < 0.01 compared to the FX 25 μM group.

To further study the molecular mechanism of G_0_/G_1_ phase cell cycle arrest induced by FX, we treated A549 and H1299 cells with FX (12.5, 25 and 50 μM) for 48 h. The expression of cell cycle-related proteins was examined by qRT-PCR and western blotting. As shown in Figure 3A,C, p21^waf1/cip1^ and p53 were upregulated in the A549 cell. H1299 cells lack p53, while its p21^waf1/cip1^ was upregulated (Figure 3B,D).

### 2.4. FX Induces Apoptosis in NSCLC Cells

We further investigated the apoptotic effects of FX in NSCLC cells. As shown in Figure 2C, the apoptosis rates in A549 cells were 26.4% ± 2.96%, 38.6% ± 7.13% and 40.6% ± 9.72% in the 12.5, 25 and 50 μM dose groups, which were much higher than the control group (1.70% ± 1.13%). H1299’s apoptosis rates were 18.1% ± 4.38%, 23.8% ± 6.51% and 27.9% ± 2.07% (12.5, 25 and 50 μM) in the presence of FX, with control group only was 0.93% ± 0.60% (Figure 2D).

### 2.5. Effects of FX on the Expression of Apoptosis-Related Genes in NSCLC Cells

We examine mRNA and protein expression of some key apoptosis regulators after FX treatment in A549 and H1299 cells. They are Bcl-2, PUMA and Fas. As shown in Figure 3A,B, PUMA and Fas were upregulated while Bcl-2 was downregulated, in a dose-dependent manner. The activities of caspase-3 and caspase-8 were also analyzed. As shown in Figure 3E,F, the activity of caspase-3 and caspase-8 was increase after FX treatment in a dose dependent manner. 

### 2.6. FX Inhibits Human NSCLC A549 Tumor Xenograft Growth in Nude Mice

**Figure 4:**
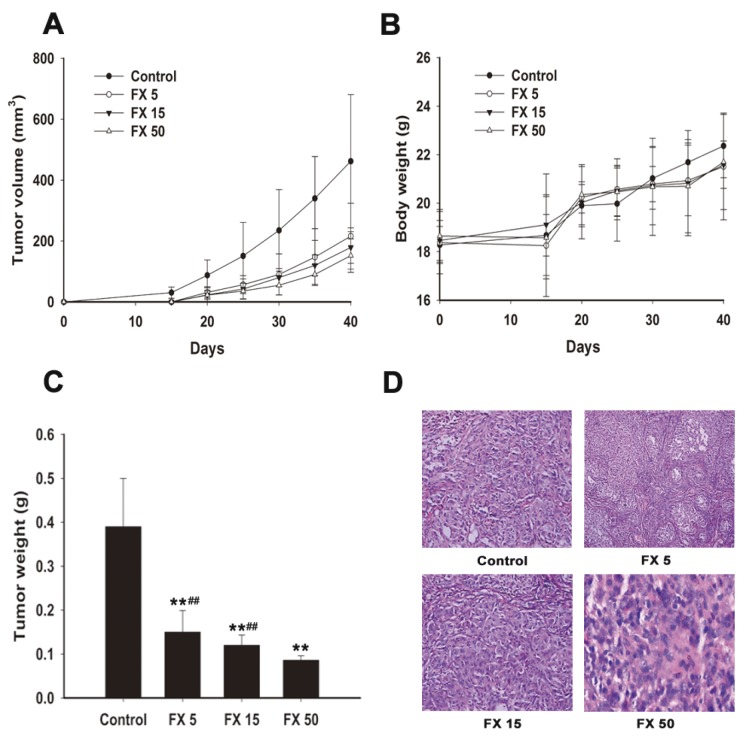
FX inhibits A549 tumor xenograft growth in vivo. The cells were subcutaneously injected into the left back of nude mice to induce tumor xenografts. Mice were gavaged with blank soybean oil, 5, 15 and 50 mg/kg FX once daily for five weeks. Tumor volumes (**A**), bodyweights (**B**) and tumor weights (**C**) were determined. Data are expressed as means ± SD (*n* = 8). ** and ^##^ denote statistically significance (*p* < 0.01) compared to the control and FX 50 mg/kg groups, respectively. (**D**) Tumor morphology in the nude mice with xenografted A549 cells was examined by hematoxylin and eosin staining. Magnification: 400×. FX 5, FX 15 and FX 50 mean 5, 15, 50 mg/kg FX, respectively.

The in vitro results demonstrate the potent anti-lung cancer activity by FX. Then, we evaluated the therapeutic efficacy of once daily oral administration of FX against human NSCLC cells A549 growing in nude mice. Five days after tumor cell inoculation, groups of mice (*n* = 8) received daily oral administrations of vehicle control or FX at doses of 5, 15, or 50 mg/kg body weight for five weeks. The weight of the solid tumors decreased significantly in FX treatment groups (Figure 4C). FX at 5, 15 and 50 mg/kg displayed potent antitumor effect with 62.0%, 69.9% and 78.1% tumor inhibition rates (Figure 4A), respectively. The greatest inhibition was found in mice receiving 50 mg/kg FX (*p* < 0.01). Importantly, by the endpoint in this study, no apparent toxic effects were observed in any of the FX treated animals. Mice body weights did not change much compared to the control group (Figure 4B) and no apparent morphological changes and metastatic nodules were observed in major organs.

Pathological analysis of tumor samples revealed that treatment with FX decreased tumor cell density and increased necrosis. The apoptotic morphological changes including cell contraction and nuclear pycnosis were observed (Figure 4D).

### 2.7. FX Induces Apoptosis In Vivo

**Figure 5:**
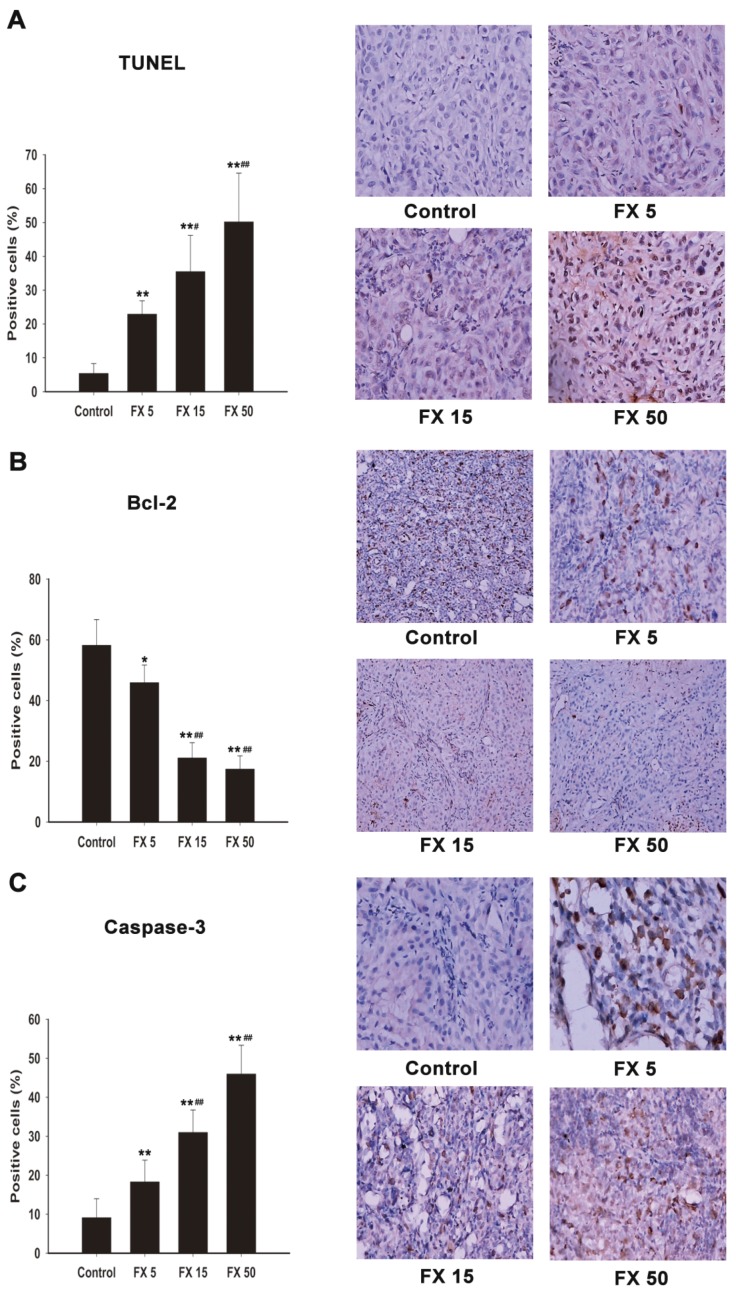
Effects of FX on the protein expression of Bcl-2 and Caspase-3 and apoptosis rates in A549 xenograft tumors. (**A**) TUNEL staining; Bcl-2 (**B**); and Caspase-3 (**C**) immunohistochemical staining. Magnification: 400×. FX 5, FX 15, FX 50 mean 5, 15 and 50 mg/kg FX respectively. Data are expressed as means ± SD (*n* = 5). * *p* < 0.05, ** *p* < 0.01 compared to the control group; ^#^ *p* < 0.05, ^##^ *p* < 0.01 compared to the FX 5 mg/kg group.

To determine the mechanisms of anti-tumor effects of FX treatments, we examined tumor apoptosis. TUNEL staining results showed that FX promoted tumor cells apoptosis significantly (Figure 5A). As shown in Figure 5B,C, Bcl-2 was downregulated and caspase-3 was upregulated, both present a dose dependency (*p* < 0.01 or *p* < 0.05). These results were consistent with the in vitro data, demonstrating that FX inhibited lung tumor by inducing tumor cell apoptosis.

## 3. Discussion

Fucoxanthin is a natural biologically active carotenoid abundant in micro- and macroalgae. FX has been shown to inhibit obesity [18,19], inflammation [20,21] and tumor growth [11,12,22,23]. However, its efficacy in NSCLC still remains to be further revealed. Here we uncovered that FX can significantly inhibit NSCLC growth and both in vitro and in vivo analyses have provided a detailed picture regarding the molecular mechanisms including cell cycle arrest and apoptosis induction. 

Using cell viability assay, we demonstrated the anti-proliferative effect of FX and its dose- and time- dependence in the NSCLC cell lines, including A549, SPC-A1, H460 and H1299, as shown in Figure 1. Our results are consistent with the previously reports that FX extracted from *Undaria pinnatifida* was effective in inhibiting the growth of A549 cells [24]. FX inhibited human colon carcinoma and HCT116 cells by cell cycle arrest during the G_0_/G_1_ phase [25]. Similarly, we found FX induced G_0_/G_1_ cycle arrest in A549 and H1299 cells significantly and dose dependently (Figure 2A,B). FX remarkably inhibited the viability of human colon cancer cell, cervical cancer and murine melanoma B16F10 cells by inducing apoptosis [26,27,28]. Using flow cytometry, we also observed that apoptosis ratios were increased after FX treatment in NSCLC cells (Figure 2C,D). Even in a low concentration, FX induced apoptosis remarkably, having shown that FX has therapeutic potential for NSCLC.

To further reveal the mechanisms of cell cycle arresting and pro-apoptotic effects after FX treatment, we performed qRT-PCR and western blotting assay. p21^waf1/cip1^ being one of the downstream genes of p53, functions as a regulator of cell cycle progression at G_1_ and S phases [29]. Consistent with previous reports [25,30], we observed that p21^waf1/cip1^ was upregulated by FX both in mRNA and protein levels (Figure 3A–D). While FX has reported to increase the ratio of cells in G_2_/M phase in MGC-803 cells by regulating the JAK/STAT signaling pathway [31]. It is probable that FX interacts with different dominating pathways in different cell lines, thus might present different cell cycle behaviors. We concluded that FX induced G_0_/G_1_ cycle arrest through up-regulating p21^waf1/cip1^ in NSCLC cells. 

Referring to apoptosis, death signaling requires clustering of Fas and finally the activation of caspase-8, which induces apoptosis [32,33]. Basically, we found Fas was upregulated significantly both in A549 and H1299 cells after the treatment of FX (Figure 3A–D). The mRNA and protein levels were changed similarly. We observed that p53 and P-p53 were upregulated with dose dependency after the treatment of FX in A549 cells (Figure 3A,C). p53 induces cell apoptosis through transcriptional-dependent and independent mechanisms by regulating genes, trans-activating the genes in both mitochondrial and death receptor pathways as well as trans repressing IGFR and Bcl-2 [34]. What’s more, PUMA is involved in apoptosis and can be induced by a variety of signals [35,36]. After activation, PUMA interacts with anti-apoptotic Bcl-2, thus regulate apoptosis by controlling mitochondrial permeability [35,37]. As shown in Figure 3A–D, PUMA was upregulated and Bcl-2 was downregulated with dose dependency (Figure 3A–D). Biochemically, caspase cascade activation and DNA fragmentation play a key role in apoptosis [38,39]. We found that FX could increase caspase-3 and caspase-8 activity remarkably (Figure 3E,F). Fas is an extrinsic factor of apoptosis while Bcl-2 and PUMA are intrinsic factors and caspase-8 plays an important role in extrinsic apoptosis [40]. Thus, we concluded that FX was able to induce apoptosis through endogenous and exogenous pathway of apoptosis in NSCLC cells and these effects were dose dependent.

After confirming the anti- proliferative and pro-apoptosis effects of FX in NSCLC cells in vitro, we wondered if FX has the above effects in vivo, too. So A549 xenograft induction was performed in nude mice. It has been illustrated that FX suppressed the growth of HeLa cells in vivo [27] and inhibited the growth of sarcoma 180 (S180) in mice significantly [41]. Similarly, FX significantly decreased the tumor volume (Figure 4A) and weight (Figure 4C) with a dose dependent behavior. We did not observe obvious body weight loss (Figure 4B) and morphological changes in major organs after FX treatment. It demonstrated that FX had a lower toxicity than traditional chemotherapy drugs. Then we performed HE staining. As shown in Figure 4D, FX caused morphological changes of necrosis and apoptosis. Furthermore, we used TUNEL staining and revealed that FX could increase apoptosis ratio remarkably with a dose dependent behavior in vivo (Figure 5A). To further reveal the pro-apoptosis mechanism, we performed immunohistochemistry analysis to detect the protein level changes of Bcl-2 and caspase-3 in tumor tissues. Consistent with previous results in vitro (Figure 3), FX dose dependently decreased Bcl-2 and increased caspase-3 (Figure 5B,C) level. Thus, we concluded that FX induced apoptosis remarkably in vivo through upregulating caspase-3 and inhibiting Bcl-2.

In summary, our study has elucidated the anti-cancer mechanisms of FX in NSCLC cells for inducing cell cycle arrest and apoptosis. In addition, we also found that FX could inhibit A549 tumor growth in nude mice. Biopsy analysis has also verified the apoptosis induction mechanism. In the future, attention should be given to establishing a more detailed understanding of the mechanisms and the synergistic antitumor effects of combined FX and other drugs. In conclusion, our study provided convincible data to merit application of FX in NSCLC drug discovery.

## 4. Experimental Section

### 4.1. Reagents and Chemicals

FX was isolated from *Laminaria japonica*, as previously described [42]. Purification from the extract was followed the method of Yan et al. [43]. The purity of FX was 100%, detected by HPLC and HPTLC, respectively. It was provided by HYDROX Co., Ltd. (Saitama, Japan) and Rongbao HighTech Co., Ltd. (Wuhan, China). The followings were purchased from Sigma (St. Louis, MO, USA): propidium iodide (PI), trypsin. Annexin V-FITC Apoptosis Detection Kits were purchased from Key Gen Biotech (Nanjing, Jiang Su, China). RNA-direct™ SYBR Green Real time PCR Master Mix kits were purchased from Toyobo Co. (Osaka, Japan). Caspase-3 and caspase-8 assay kits were purchased from BD Biosciences (San Diego, CA, USA). Cell counting kit-8 kits were purchased from Dojindo Laboratories (Kumamoto, Japan). Anti-p53 and anti-phosphated p53 antibodies were purchased from Cell Signaling Technology (Danvers, MA, USA). Anti-Bcl-2, anti-p21^waf1/cip1^, anti-PUMA, anti-FAS, anti-β-actin antibodies and horseradish peroxidase-conjugated secondary antibodies were from Santa Cruz Biotechnology, Inc. (Santa Cruz, CA, USA). Polyclonal Antibodies against p53, Bcl-2, Caspase-3 were from Boster Biological Technology, Ltd. (Wuhan, Hubei, China). ECL was purchased from Pierce Biotechnology (Waltham, MA, USA). Tunnel staining in situ cell death detection kit was purchased from Roche (Minneapolis, MN, USA). Immunohistochemistry kits for rabbit and mouse antibodies were purchased from Gene Tech (Shanghai, China).

### 4.2. Cell Culture

All NSCLC cell lines (A549, H460, SPC-A1 and H1299) were obtained from Cell Bank of the Chinese Academy of Sciences Committee Type Culture Collection (Shanghai, China) and cultured in RPMI-1640 medium (Gibico, Grand Island, NY, USA) with 10% fetal bovine serum, 100 U/mL penicillin and 100 pg/mL streptomycin (Gibico, Grand Island, NY, USA) in a 5% CO_2_ and 95% air incubator at 37 °C. The cells were passaged by 0.125% trypsin-EDTA when they reached 80% confluence.

### 4.3. Cell Viability

Cells were treated with different concentrations of FX for 24, 48, or 72 h before cytotoxic activity was evaluated by the Cell counting kit-8 (Dojindo Laboratories, Kumamoto, Japan) and the absorbance was measured at 450 nm.

### 4.4. Cell Cycle Analysis

Cells (5 × 10^5^/well) were seeded into 6-well plates and exposed to FX at various concentrations (12.5, 25 and 50 μM) for 48 h and then harvested and washed with PBS, fixed in 70% ethanol at 4 °C. Staining went along with PBS containing 1 mg/mL RNaseA and 50 μg/mL PI in the dark at room temperature for 20 min. The cell cycle was measured using FACSCalibur^®^ flow cytometer (BD, Franklin Lakes, NJ, USA).

### 4.5. Measurement of Apoptosis by Annexin V-FITC/PI Staining

Flow cytometry was used to quantitatively detect the apoptotic rate. Cells (5 × 10^5^ /mL) were seeded into 6-well plates and exposed to FX at various concentrations (12.5, 25 and 50 μM) for 48 h and then harvested and washed with phosphate buffered saline (PBS). Staining went along with bonding buffer containing Annexin V-FITC in the dark at room temperature for 10 min and then added PI in the dark 4 °C for 10 min. The apoptotic cells were analyzed with FACS Calibur™ flow cytometer. 

### 4.6. Real-Time Quantitative PCR

After 48 h treatment of FX at the concentration of 12.5, 25 and 50 μM, total RNA was isolated with Trizol reagent (Invitrogen, Grand Island, NY, USA) following the manufacturer’s instructions. The RNA samples were reverse-transcribed using RNA-direct™ SYBR Green Realtime PCR Master Mix kit (Toyobo, Osaka, Japan). RNA amplification and quantitative determination were carried out in a SLAN-96P real-time PCR system (Hongshi Medical Technology Company, Ltd., Shanghai, China). Cycling conditions were performed as follows: 1 min at 50 °C, 2 min at 94 °C, then 40 cycles of 15 s at 94 °C, 15 s annealing, 45 s at 72 °C and then 10 min at 72 °C. β-actin mRNA levels were also quantified in each sample and were used as a normalization control. The comparative C_T_ method was used to analyze the PCR data.

### 4.7. Western Blot

Cells were lysed with ice-cold lysis buffer (50 mM Tris-HCl, pH 7.4, 100 mM NaCl, 1 mM EDTA, 20 mM NaF, 3 mM Na_3_VO_4_, 1 mM PMSF) for 30 min on ice. Cell lysates were then collected after centrifugation at 12,000 rpm for 5 min at 4 °C. Equal amount of sample proteins were separated on 10% SDS-PAGE gels and transferred onto PVDF membranes (Millipore, Billerica, MA, USA). After blocking with 5% defatted milk powder in TBST buffer (10 mM Tris, pH 7.5, 150 mM NaCl and 0.05% Tween 20) for 45 min at room temperature. The protein samples were then immunoblotted with primary monoclonal antibodies. They were mouse monoclonal antibody against phosphate-p53; rabbit polyclonal antibodies against p53, Bcl-2, Fas, p21^waf1/cip1^ and β-actin; and goat polyclonal antibodies against PUMA. After incubating for 1 h at room temperature with secondary antibodies (horseradish peroxidase-conjugated goat anti-mouse IgG, goat anti-rabbit IgG and rabbit anti-goat IgG), antigens were visualized using enhanced chemiluminescence using ECL according to the manufacturer’s instructions. 

### 4.8. Caspase-3 and Caspase-8 Activity Assay

Enzymatic activities of caspase-3 and -8 were measured using caspase-3, caspase-8 colorimetric assay kits, according to the manufacturer’s procedure. After exposure to 12.5, 25 and 50 μM FX for 48 h, control or treated cells were lysed in 50 μL chilled cell lysis buffer. Supernatant was collected by centrifugation at 11,500 rpm for 10 min at 4 °C. 50 μL of 2× Reaction Buffer/DTT Mix was added and 1 μL of caspase-3 inhibitor (DEVD-fmk) or caspase-8 inhibitor (IETD-fmk) was incubated with 50 μL of supernatant from a sample on ice for 30 min. 5 μL of 1 mM capase-3 or caspase-8 substrate (DEVD-pNA) was added and incubated at 37 °C for 1 h in a water bath. The samples were analyzed at 405 nm in a microplate reader (Awareness Technology Inc., Palm City, FL, USA). The activities of caspase-3 and caspase-8 were calculated according to the calculated formula in the assay kit.

### 4.9. Antitumor Activity Study of FX In Vivo

Animal experiments were approved by the Institutional Animal Ethical Committee of Huazhong University of Science and Technology. Female and male (1:1 ratio) BALB/c nude mice (18–20 g; 6–8 weeks of age) were purchased from the Vital River Laboratories (Beijing, China) and received care in compliance with the guidelines proposed by Institutional Animal Care and Use Committee. All the mice were injected in the right flank subcutaneously with A549 cells (2 × 10^6^ per mouse). After five days, tumor-bearing mice were randomly divided into four groups (*n* = 8, female: male = 1:1). The vehicle control group was gavaged with blank soybean oil. In the FX treatment groups, FX was administered at a dose of 5, 15 and 50 mg/kg each day orally for five weeks. All groups were observed for 40 days. Mice were weighed and tumors were measured using calipers every five days. Tumor volumes were determined by measuring the length (*L*) and the width (*W*) and calculated as *V* = π/6 × *L* × *W*^2^. The animals were sacrificed on Day 41 when subcutaneous tumors were isolated and weighed to calculate the tumor inhibition rate. Tumor inhibition rate (%) = (1 − tumor tissue weight of treatment group /tumor tissue weight of control group) × 100%. Tumor tissues were fixed in 10% neutral formaldehyde solution, embedded in paraffin and sliced into 4–5 μm sections for hematoxylin/eosin, immunohistochemical staining and TUNEL detection, the other parts are stored frozen (−80 °C). 

### 4.10. Immunohistochemistry Analysis

The immunohistochemical staining of tumor Bcl-2 and Caspase-3 expression followed the manufacturer’s instructions. The images were captured under a CX21 microscope (Olympus, Shinjuku, Tokyo, Japan). 

### 4.11. TUNEL

To detect apoptotic cells in tumor tissues, the TUNEL assay was performed following the manufacturer’s protocol. Cell nuclei with brown staining were defined as TUNEL-positive nuclei. To quantify TUNEL-positive cells, the number of positive cells was counted in five random areas at 400× magnifications.

### 4.12. Statistics

Data were expressed as means ± S.D. from at least three independent experiments. Statistical analysis was performed using SPSS 19. *p* Values were calculated using Student’s *t* test (alpha level: 0.05, two-tailed). The differences between the groups were considered significant at *p* values less than 0.05.

## 5. Conclusions

Fucoxanthin inhibited NSCLC cell growth both in vitro and in vivo. FX induced cell cycle arrest and apoptosis by upregulating p53, p21^waf1/cip1^, PUMA and Fas and downregulating Bcl-2. Our results provide the possibility that FX will become a potential drug for patients with NSCLC.

## Figures and Tables

**Table 1:** IC_50_ of fucoxanthin on proliferation of four NSCLC cell lines.

Cells	Time (h)	IC_50_ (μM)
A549	24	51.6 ± 3.89
	48	28.8 ± 1.99
	72	14.32 ± 0.21
H460	24	69.1 ± 4.70
	48	40.9 ± 8.09
	72	17.0 ± 1.42
SPC-A1	24	60.1 ± 2.39
	48	38.8 ± 2.08
	72	15.0 ± 5.25
H1299	24	81.5 ± 4.72
	48	56.4 ± 1.54
	72	23.7 ± 1.31

Data are presented as means ± SD (*n* = 3).

**Table 2:** Cell cycle distribution of A549 and H1299 cells after fucoxanthin treatment for 48 h.

Group	A549 Cells			H1299 Cells		
	Cell Cycle Percentage (%)			Cell Cycle Percentage (%)		
	G_0_/G_1_	S	G_2_/M	G_0_/G_1_	S	G_2_/M
Control	45.7 ± 5.33	48.0 ± 10.1	6.28 ± 5.24	39.0 ± 6.51	45.6 ± 3.44	7.69 ± 6.16
FX 12.5 μM	61.0 ± 4.46 *	29.7 ± 7.82	9.4 ± 4.02	51.3 ± 4.85	31.2 ± 7.27 *	17.5 ± 10.3
FX 25 μM	61.3 ± 3.95 *	33.7 ± 1.2	5.01 ± 2.76	56.2 ± 4.62 *	35.1 ± 5.63	8.69 ± 1.1
FX 50 μM	66.3 ± 4.55 **	30.2 ± 1.41 *	3.55 ± 3.18	57.3 ± 6.42 *	30.3 ± 1.04 **	12.3 ± 5.38

Data are presented as means ± SD (*n* = 3). * *p* < 0.05 vs. the control group, ** *p* < 0.01 vs. the control group.
